# The modelled impact of increases in physical activity: the effect of both increased survival and reduced incidence of disease

**DOI:** 10.1007/s10654-017-0235-1

**Published:** 2017-03-03

**Authors:** Oliver T. Mytton, Marko Tainio, David Ogilvie, Jenna Panter, Linda Cobiac, James Woodcock

**Affiliations:** 1 MRC Epidemiology Unit and UKCRC Centre for Diet and Activity Research (CEDAR), University of Cambridge School of Clinical Medicine, Box 285, Cambridge Biomedical Campus, Cambridge, CB2 0QQ UK; 2 Systems Research Institute, Polish Academy of Sciences, Newelska 6, 01-447 Warsaw, Poland; 3 Centre for Health Policy, School of Population and Global Health, University of Melbourne, 207 Bouverie Street, Melbourne, Carlton, VIC 3053 Australia

**Keywords:** Physical activity, Modelling, Lifetable, Survival, Disease burden

## Abstract

Physical activity can affect ‘need’ for healthcare both by reducing the incidence rate of some diseases and by increasing longevity (increasing the time lived at older ages when disease incidence is higher). However, it is common to consider only the first effect, which may overestimate any reduction in need for healthcare. We developed a hybrid micro-simulation lifetable model, which made allowance for both changes in longevity and risk of disease incidence, to estimate the effects of increases in physical activity (all adults meeting guidelines) on measures of healthcare need for diseases for which physical activity is protective. These were compared with estimates made using comparative risk assessment (CRA) methods, which assumed that longevity was fixed. Using the lifetable model, life expectancy increased by 95 days (95% uncertainty intervals: 68–126 days). Estimates of the healthcare need tended to decrease, but the magnitude of the decreases were noticeably smaller than those estimated using CRA methods (e.g. dementia: change in person-years, −0.6%, 95% uncertainty interval −3.7% to +1.6%; change in incident cases, −0.4%, −3.6% to +1.9%; change in person-years (CRA methods), −4.0%, −7.4% to −1.6%). The pattern of results persisted under different scenarios and sensitivity analyses. For most diseases for which physical activity is protective, increases in physical activity are associated with decreases in indices of healthcare need. However, disease onset may be delayed or time lived with disease may increase, such that the decreases in need may be relatively small and less than is sometimes expected.

**Electronic supplementary material:**

The online version of this article (doi:10.1007/s10654-017-0235-1) contains supplementary material, which is available to authorized users.

## Introduction

People who undertake regular physical activity tend to experience better health and live longer [1–3]. Many countries therefore aim to promote physical activity to improve population health [4–6]. Some go further and assume that increases in it will also reduce ‘need’ for health and social care [4, 7–9]. The implicit logic appears to be that improving the population distribution of a risk factor such as physical activity will reduce the incidence rate of disease, thereby resulting in fewer incident cases and fewer people living with disease, thereby reducing need for healthcare.

### Effect of physical activity on disease

**Fig. 1:**
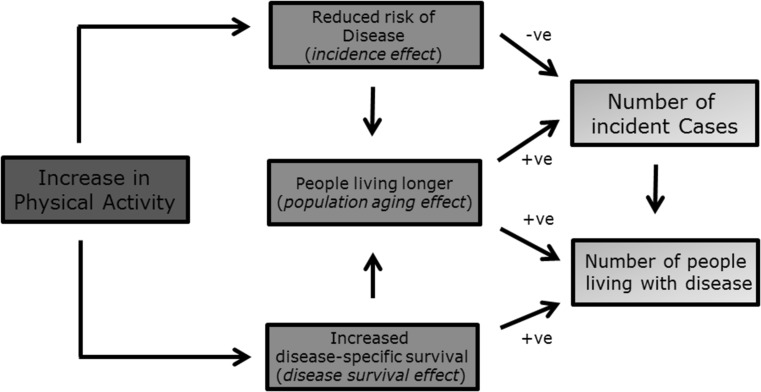
How increases in physical activity may affect the number of incident cases of and people living with cardiovascular disease

However, increases in physical activity may affect the number of people living with disease by several pathways, not all of which will act to reduce the number of people living with disease (see Fig. 1). First a reduction in relative risk, arising from an increase in physical activity, will lead to a reduction in the incidence rate of disease. All other things being equal, this will result in fewer incident cases of diseases and consequently fewer people living with disease. We term this the ‘incidence effect’.

However there is a second opposing effect, which we will term ‘population aging’ (shown in yellow in Fig. 1). This is an increase in the number of older people because of reduced mortality, resulting from reduced risk of disease or increased (disease-specific) survival. As the incidence rate of many chronic diseases increases with age [10–12], this will result in an increase in the absolute number of incident cases, and therefore also in the number of people living with disease.

A third effect may also occur, which we will term the ‘disease survival effect’. Physical activity may increase disease-specific survival, for example it is used as a treatment for some diseases (e.g. ischaemic heart disease) [13]. The average duration of disease survival will increase, resulting in more people living with disease. It will also contribute to population aging. Consequently, when considering these later two effects (‘population aging’ and ‘disease survival’), it is no longer clear whether and the extent to which increases in physical activity will be associated with reductions in the number of incident cases or the number of people living with disease.

From an individual (or population) perspective all three effects are a form of ‘health gain’. Respectively they result in reduced risk of disease onset, increased life expectancy, increased disease-specific life expectancy (and likely an associated reduction in disease severity). However, our interest is in exploring their cumulative effect, at the population-level, on need for health and social care, specifically incident cases and people living with disease.

### Summary of existing research

Whilst we think the question is intrinsic to modern public health practice, particularly in the UK where there is an expectation that preventive health services should reduce pressure on health and social care [4, 7, 9], surprisingly little research has explored these issues. There is an existing literature concerned with disease expansion and compression, respectively referring to an increase and a decrease in the mean duration an individual person lives with disease [13–15]. The focus of this literature is understanding how health and life expectancy have evolved in past, or may evolve in the future [13, 14, 16], rather than understanding the effect of changes in individual risk factors on the number of individuals with disease.

A few observational studies have tested the association between physical activity and healthcare utilisation, but such studies, particularly when cross-sectional, do not adequately account for disease being postponed until after the period of observation [15, 17–20]. Studies that make use of lifetable modelling (and which use data from observational studies) can address this limitation, but have generally described the effects at the individual rather than the population level [19–24]. These studies have tended to focus on single diseases, often cardiovascular disease, [19, 20, 22] so may not adequately consider how one disease may affect another disease (e.g. changes in dementia incidence may be brought about by reduced incidence of and increased survival from cardiovascular disease). They report only one measure of healthcare need, average years lived with disease or disability. This measure does not consider how many people develop disease (i.e. do a few people live with disease for a long time, or many people for a short time), which may have implications for healthcare resources.

Understanding the effect of increases in physical activity on the indices of disease burden is also important for health impact modelling, an increasingly important tool that seeks to estimate the health benefit from preventive interventions [25]. Whilst some modelling methods (e.g. micro-simulation and multi-state life table) can make allowance for changes in life expectancy, such techniques are often not employed when undertaking physical activity health impact modelling [26–28] or estimating the burden of disease attributable to insufficient physical activity [29, 30].

### Study aims

The aim of this paper is to contribute to a richer understanding of how physical activity may affect disease in a population as it relates to need for health and social care (incident cases and people living with disease), making allowance for changes in longevity. While our focus is physical activity many of the principles that the paper outlines will apply to other risk factors. We are primarily interested in diseases for which regular physical activity is protective and do not consider in detail diseases whose incidence is independent of physical activity (e.g. some cancers) but whose incidence rises with age.

## Methods

### Model description

**Fig. 2:**
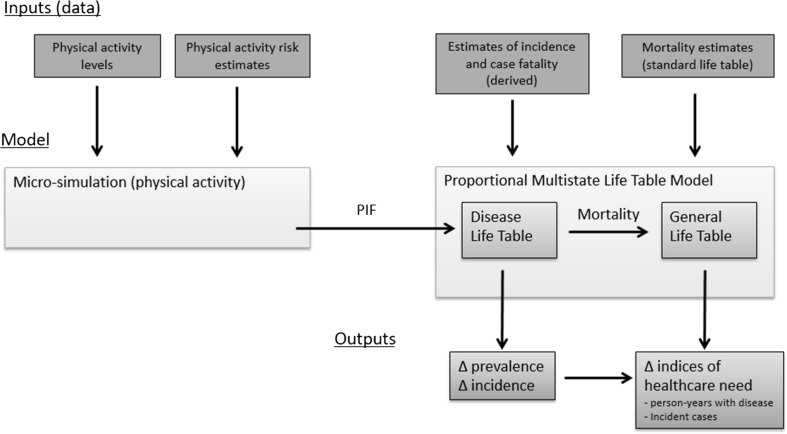
Schematic outline of the model. *PIF* = potential impact fraction

We developed a hybrid micro-simulation life table model (Fig. 2) to describe the effects of changes in physical activity within the English adult population on survival and indices of need. This used two modelling processes: (1) micro-simulation that described the effect of changes in physical activity on disease risk at the individual level, from which population impact fractions for disease incidence and disease case fatality were derived; and (2) a proportional life table model that described the effect of changes in incidence and case fatality on prevalence and survival for each disease. From this estimates of changes in the indices of need were made. Further information is given in the methods supplement.

1. The micro-simulation model

We simulated a population of 8118 adults, representative of the English adult (aged 16 years and over) population in terms of age, sex and physical activity level. Each individual’s physical activity level could change independently and was related to their disease risk. Physical activity level was measured in marginal MET-hours, a product of the intensity and duration of physical activity [27, 31]. Given the evidence of a non-linear relationship between physical activity and disease risk, and following the approach used by others, we assumed that disease risk was log linearly associated with a power transformation of the physical activity exposure [31].

Changes in physical activity and consequent change in disease risk for an individual were modelled by shift along the physical activity disease curve. Potential impact fractions, a measure of change in average disease risk, were estimated by a weighted sum of the ratio of the relative risk observed under different scenarios of increases in physical activity compared to baseline (i.e. physical activity levels are unchanged). This is a standard measure, which is similar to a population attributable fraction, and is used to estimate the change in health status of a population due to a change in the distribution of a risk factor within a population [32, 33]. Allowance was made for a delay between physical activity and its effect on disease risk.

2. Proportional multistate life table model

We used a proportional multi-state life table model, consisting of two parts: a general life table model, and a set of disease life tables [34]. This approach has been adopted by others to model the effect of physical activity [22, 35], or other risk factors, on health [21, 36].

Briefly, the general life table consisted of two states (alive and dead) and described the probability of dying at any given age in the subsequent year. The general life table was used to describe survival of a cohort from birth to death, and estimated the number of people alive. Each disease life table only tracked events related to a single disease, and consisted of three states (alive without disease, alive with disease, dead). Transition hazards (incidence and case fatality) were used to estimate the probability of moving between states in any given year of life. We assumed no disease remission. The disease life tables were used to estimate disease prevalence and disease-specific mortality by age and sex.

Under each scenario a new set of transition hazards (incidence and case fatality) for each disease was calculated by multiplying the baseline transition hazards by potential impact fraction. This in turn led to a new estimate of disease-specific mortality. Changes in each disease-specific mortality fed into the general life table altering the probability of dying, thus allowing us to model changes in survival (population aging) that results from changes in disease incidence and disease-specific survival for the six diseases.

### Diseases

We included diseases if: (a) they were important causes of morbidity or mortality; (b) there was wide consensus that physical activity reduced incidence of that disease; and (c) estimates of the effect of physical activity on incidence of that disease could be extracted from published meta-analyses. Diseases included were ischaemic heart disease, stroke, type 2 diabetes, dementia, colon cancer and breast cancer [3, 30, 46]. We assumed that physical activity affected the incidence of all six diseases and that it affected survival (case fatality) of three diseases (ischaemic heart disease, colon cancer and breast cancer).

### Outcomes

We chose two primary indices of need that may reflect healthcare utilisation: number of people living with disease and number of incident cases [37, 38]. To ensure we measured disease that was postponed until later life, we followed a cohort (n = 100,000) from birth to death (or 100 years of age). Measuring these indices across the life of the cohort gave two outcomes: person-years lived with disease, and total incident cases. We prefer the term ‘need’ in preference to ‘burden’ which is less precise and may also encompass years of life loss [30, 39].

We estimated the person-years lived with disease by summing the product of the age-specific prevalence (taken from the disease life table) and the number of people alive at each age (taken from the general lifetable). We estimated total incident cases by summing the product of the age-specific disease incidence (taken from the disease life table) and the number of people alive at that age. We then estimated percentage change under the scenario being studied (relative to baseline) for these two outcomes.

The former outcome may be an important indicator of need for healthcare [37, 38, 40] where significant resources are required throughout the course of the disease (e.g. type 2 diabetes or dementia). The latter may be an important indicator of need where significant resources are required around the time of diagnosis (e.g. cancer).

To compare our estimates with measures that do not make allowance for increasing life expectancy, we used comparative risk assessment (CRA) methods to estimate the change in person-years with disease, by summing the product of age-specific prevalence (at baseline), the number of people alive (at baseline) and the potential impact fraction [41, 42]. We estimated the percentage change relative to baseline. We term this metric ‘person-years with disease (unchanged life expectancy)’.

The estimates of potential impact fraction used in the lifetable model and when using comparative risk assessment methods were the same. The observed differences between the two methods thus reflected the different way that these two methods simulated changes in survival and the pathways they explicitly modelled. Comparative risk assessment models consider only the incidence effect, whereas proportional multistate lifetable additionally consider the population aging effect and the disease survival effect (Fig. 1).

We also estimated the change in life expectancy for each scenario using the general life table.

### Scenarios

We explored two scenarios. First, ‘meeting guidelines’, in which all adults met the UK adult physical activity guidelines (150 min of moderate-to-vigorous physical activity (MVPA) per week) [43]. We assumed this was achieved by walking for 150 min on flat ground at 3 mph, which is likely to be the most feasible way for the population to meet this goal. This is equivalent to 5.75 marginal MET-hours per week [44]. Those individuals who were already undertaking at least this amount did not change their physical activity level, all other individuals increased their physical activity level to 5.75 marginal MET-hours.

Second, ‘Shift’, in which we assumed that all adults, irrespective of their current physical activity level, increased their physical activity by 5.75 marginal MET-hours. We also modelled the effect of a shift of half (2.875 marginal MET-hours, equivalent to 75 min walking or similar MVPA per week) and 50% more (8.625 marginal MET-hours, equivalent to 225 min of walking or other MVPA per week) than this.

Each scenario is compared to baseline, i.e. no increase in physical activity above current physical activity levels.

### Data

We used the following sets of data: data on physical activity; data describing the relationship between physical activity and disease; estimates of transition hazards (incidence and case fatality) for the disease life tables; and estimates of mortality for the general life table. We sought data that were representative of the English population.

Estimates of physical activity level by age and sex were derived from the Health Survey for England 2012, which incorporated the International Physical Activity Questionnaire (IPAQ) [45]. Physical activity level was estimated by summing the product of weekly duration of activity (in hours) and the intensity of activity (measured in marginal MET) for each activity reported. Estimates of intensity were taken from Ainsworth’s Compendium of physical activity [44].

Estimates of the association between physical activity and the outcome of interest were taken from meta-analyses of observational studies or randomised controlled trials [46–55]. We used adjusted estimates of relative risk to describe the un-confounded association between physical activity and disease risk.

Estimates of transition hazards (incidence and case fatality) were made using DISMOD II v1.05 (World Health Organisation, 2001–2009) [56], based on routine data or other large studies in the UK [11, 12, 57–61].

We used the interim lifetable for England for the years 2010-2012 [62] to parameterise the general lifetable of our model.

### Uncertainty and sensitivity analyses

We estimated 95% uncertainty intervals (2.5th to the 97.5th percentile) from 5000 iterations of a Monte Carlo analysis. For each iteration a random value was drawn from the described distribution for each parameter. We modelled uncertainty for three sets of parameters: the power transformation describing the relationship between physical activity and risk; the association of physical activity with relative risk of disease incidence; the association of physical activity with relative risk of case fatality.

We also undertook sensitivity analyses to examine the effect of changes to the model structure or parameters on the primary outcomes. Parametric uncertainty was tested by constructing tornado plots for the two primary outcomes and for change in life expectancy for each of the six diseases.

We examined structural uncertainty by making changes to the model structure (omitting, adding or changing parts of the model). We tested the following changes. First we assumed that physical activity did not affect cancer survival, to reflect uncertainty about whether physical activity has a causal role in cancer survival. The association between physical activity and survival after incident colon or breast cancer is reported in observational studies [47, 55] and could be due to confounding by indication (i.e. that people who are able to be physically active are healthier because they have a less aggressive cancer) [55, 63]. Second we assumed that physical activity reduced the incidence of other cancers (lung, prostate and pancreatic). Whilst not incorporated into some physical activity guidelines [1, 3, 43], associations between physical activity and reduced incidence of these cancers has been consistently observed [64], and our initial work suggested that our model might be under-estimating the effect of physical activity on all-cause mortality. Third we assumed that there was no lag between physical activity and its effect on disease risk. There is an absence of evidence about the length of lags, and we wanted to understand the effect that modelling lags was having on the overall picture. Fourth we assumed that only walking, sport and recreational physical activity contribute to physical activity levels. This reflects current epidemiological studies of physical activity and disease, which predominantly considers either leisure time physical activity or walking, and thus excluded domestic, transport and occupation activity.

## Results

Under the ‘meeting guidelines’ scenario estimated life expectancy (at birth) increased by 95 days (95% uncertainty intervals: 68–126 days), or 89 days for men (60–123 days) and 101 days for women (75–131 days).

**Fig. 3:**
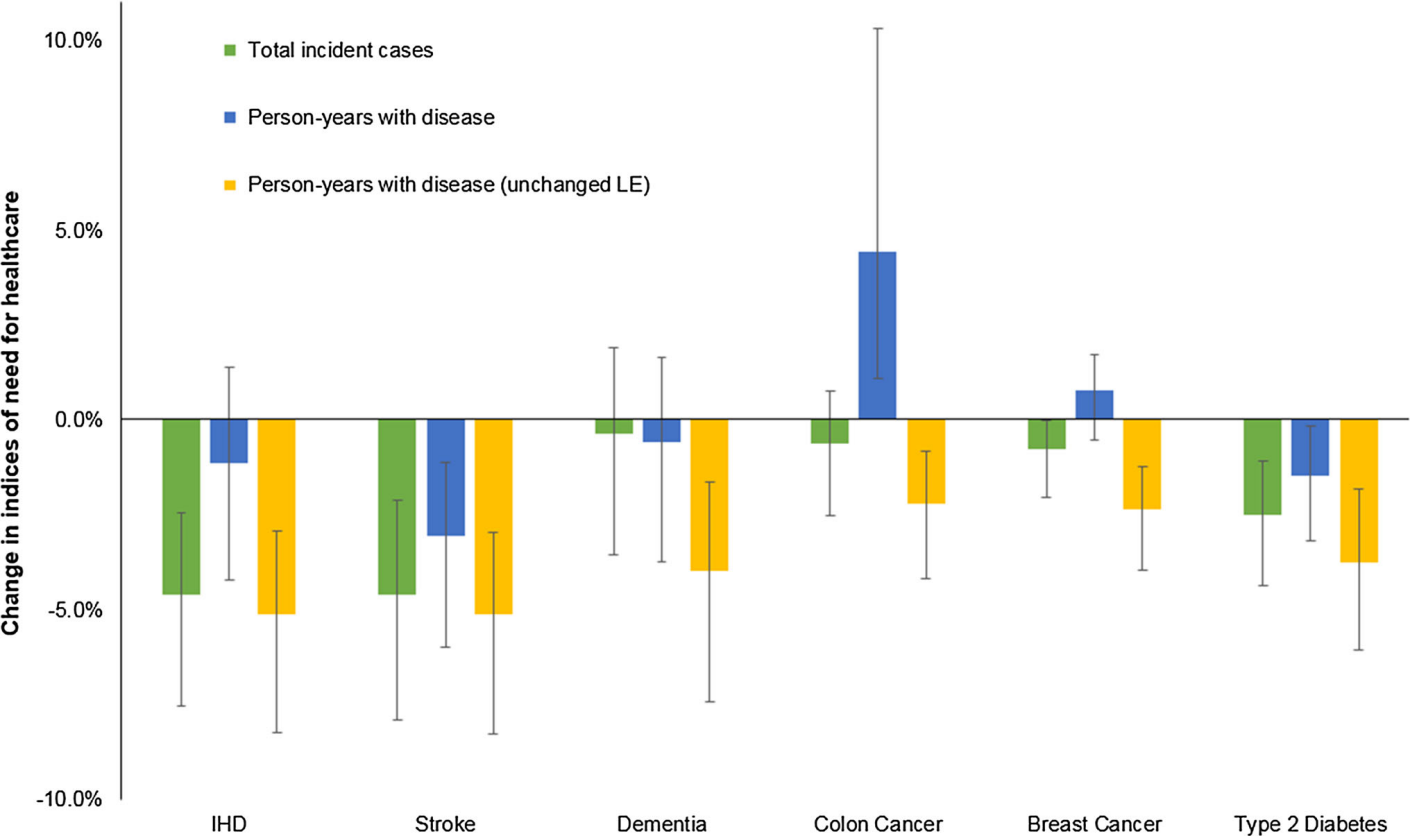
Effect of meeting physical activity guidelines on the change in indices of need. *IHD* ischaemic heart disease, *LE* life expectancy; person-years with diseases (unchanged LE) assumes that LE (life expectancy) is held constant at the baseline value—these estimates were made using comparative risk assessment methods

Changes in person-years with disease and total incident cases for this scenario are shown in Fig. 3. Person-years lived with disease decreased for ischaemic heart disease, stroke, type 2 diabetes and dementia, and increased for colon cancer (uncertainty intervals not including zero) and breast cancer (uncertainty intervals including zero). The decreases observed for ischaemic heart disease and dementia were small (with uncertainty intervals that included zero). Total incident cases decreased for all six diseases, although the 95% uncertainty intervals included zero for dementia and colon cancer.

Estimates of the decrease in person-years lived with disease were considerably smaller than estimates made using comparative risk assessment methods (Fig. 3). The differences were particularly marked for IHD, dementia, colon cancer and breast cancer.

Estimates of the change in mean age of onset (amongst those who develop disease) are shown in Table 1. For dementia, colon cancer and breast cancer the mean age of onset increased (i.e. was later). For IHD, stroke and type 2 diabetes the mean age of onset decreased.

**Table 1:** Change in mean age of disease onset and percentage of cases prevented for the ‘meeting guidelines’ scenario

	Change in mean age of onset (days)	Percentage of cases prevented (%)
IHD	−6.9 (−39.9 to 27.3)	**4.6 (2.4 to 7.6)**
Stroke	15.2 (−40.5 to 75.2)	**4.6 (2.1 to 7.9)**
Type 2 diabetes	**−39.8 (−69.1 to −14.1)**	**2.5 (1.1 to 4.4)**
Dementia	**133 (97.4 to 173)**	0.4 (−1.9 to 3.6)
Breast cancer	**73.6 (27.9 to 115)**	0.8 (0.0 to 2.0)
Colon cancer	**52.9 (18.5 to 88.9)**	0.6 (−0.8 to 2.5)

Meeting guidelines scenario assumes that all adults who are not presently doing 5.75 marginal MET-hours of physical activity (equivalent to 150 min of walking per week) increase their physical activity to 5.75 marginal MET-hours, the physical activity level of adults who are doing more than 5.75 marginal MET-hours per week is unchanged; *LE* life expectancy, *IHD* ischaemic heart disease; bold type indicates that the uncertainty intervals do not overlap with zero

**Fig. 4:**
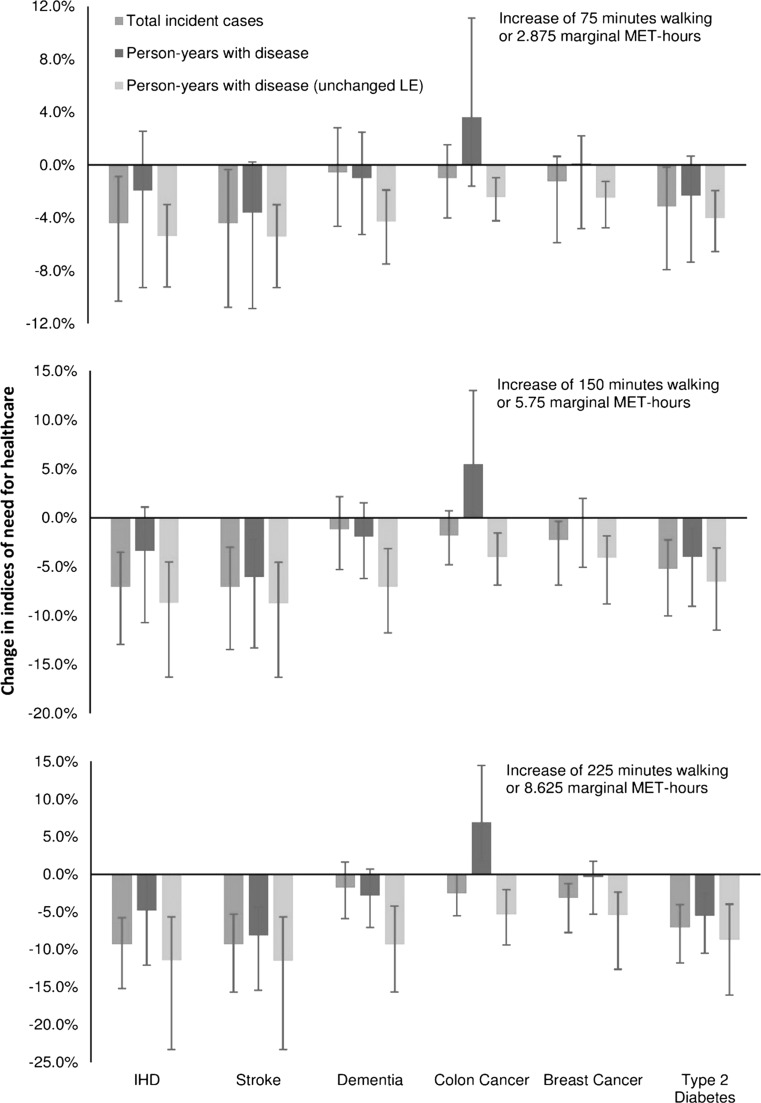
Estimate of the effect of a ‘shift’ in physical activity levels on changes in indices of need. *IHD* ischaemic heart disease; *LE* life expectancy; person-years with diseases (unchanged LE) assumes that LE (life expectancy) is held constant at the baseline value—these estimates were made using comparative risk assessment methods; three scenarios represent a ‘shift’ in physical activity whereby physical activity increases for everyone by the specified amount

Results for the shift scenarios (increases in physical activity of either 75, 150 or 225 min) showed a similar pattern, although the absolute changes were different (Fig. 4). The one noticeable difference between the ‘shift’ and ‘meeting guidelines’ scenarios was that the estimated change in person-years lived with breast cancer changed from being a small decrease (under the ‘shift’ scenario) to a small increase (under the ‘meeting guidelines’ scenarios), although for both scenarios the uncertainty interval included zero.

**Fig. 5:**
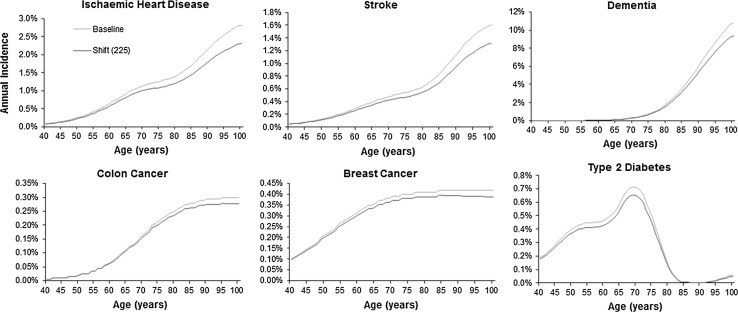
Incidence of the six diseases by age comparing baseline with a scenario of everyone doing an additional 225 min of moderate intensity physical activity. Shift (225) represents a scenario of everyone doing an additional 8.675 marginal MET-hours of physical activity per week, equivalent to an additional 225 min of additional moderate physical activity at 3.3 MET, e.g. walking at 3 mph on level ground

**Fig. 6:**
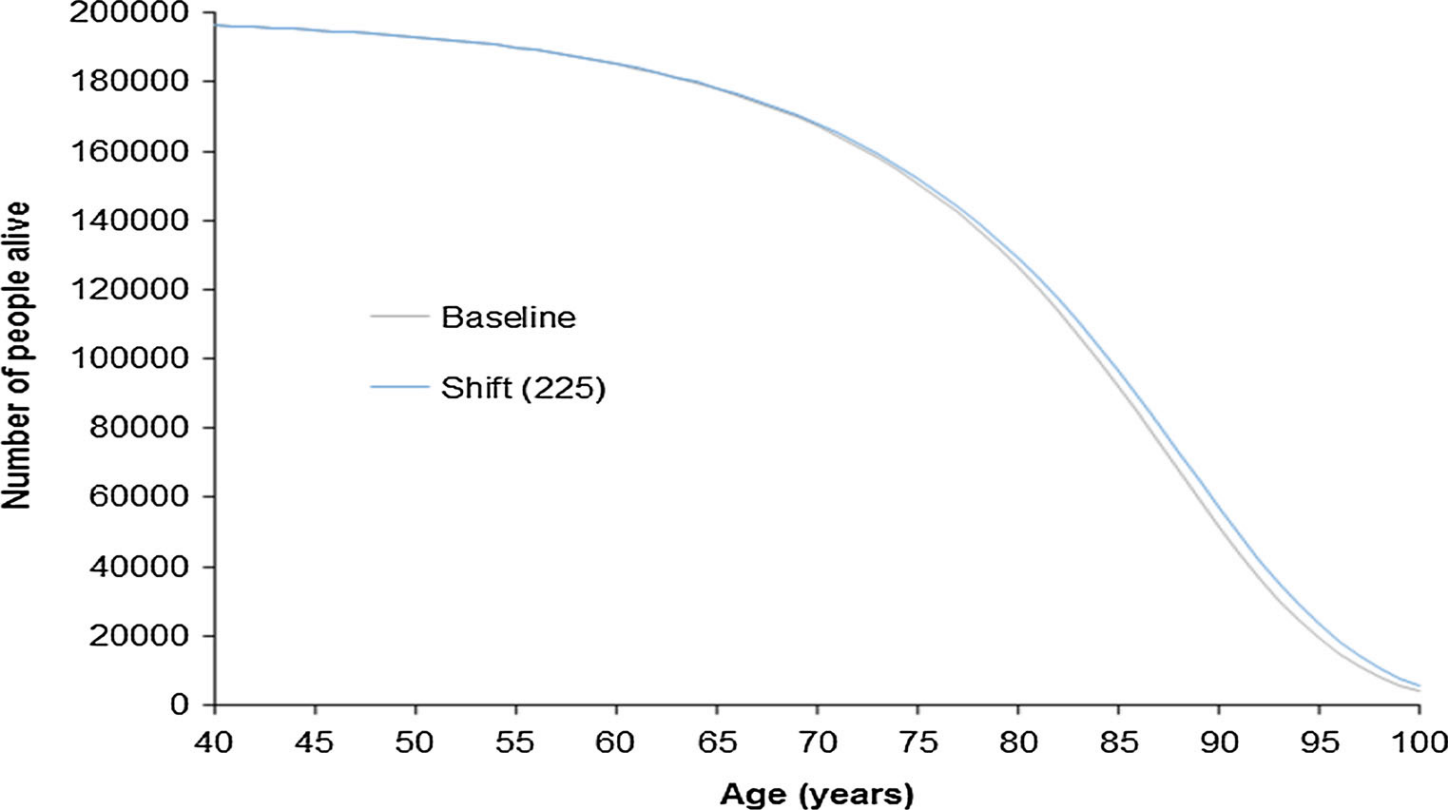
Number of people alive by age from an initial cohort of 100,000 women and 100,000 men; comparing baseline with a scenario of everyone doing an additional 225 min of moderate physical activity. Shift (225) represents a scenario of everyone doing an additional 8.675 marginal MET-hours of physical activity per week, equivalent to an additional 225 min of additional moderate physical activity at 3.3 MET, e.g. walking at 3 mph on level ground

**Fig. 7:**
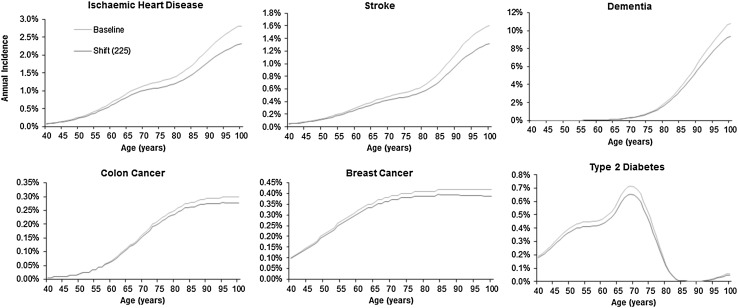
Number of people living with six different diseases by age from an initial cohort of 100,000 women and 100,000 men; comparing baseline with a scenario of everyone doing an additional 225 min of moderate physical activity. Shift (225) represents a scenario of everyone doing an additional 8.675 marginal MET-hours of physical activity per week, equivalent to an additional 225 min of additional moderate physical activity at 3.3 MET, e.g. walking at 3 mph on level ground

Graphs of survival, disease incidence and number alive with disease by age are shown for the scenario with the greatest effect (‘shift’ of 225 min) in order to highlight the pattern of change (Figs. 5, 6, 7). These show that an increase in physical activity is associated with a decrease in the incidence of each disease (Fig. 5) and a rightwards shift of the survival curve (Fig. 6). The number of people living with disease by age is shown in Fig. 7. For some diseases (e.g. stroke and type 2 diabetes) the curve representing increased physical activity is flatter, for other diseases there is a rightward shift in the curve (e.g. breast cancer) or a combination of a rightward shift and flattening (e.g. ischaemic heart disease and dementia). For colon cancer the curve shifts to the right and has a higher peak.

### Sensitivity analyses

Tornado plots showing the effect of parametric uncertainty on change in person years with disease, incident cases and life expectancy are shown in the supplementary results (Figures A2–A4). Of note, changes in the values of three parameters (association between physical activity and dementia, power transformation describing relationship between physical activity and risk, association between physical activity and ischaemic heart disease fatality) within the reported range of uncertainty, altered the estimate of change in person-years lived with dementia and of the change in total incident cases from a decrease to an increase. Similarly a stronger association of physical activity with relative risk of colon cancer incidence, altered the estimate of change in incident cases from a decrease to an increase.

The effect of different structural configurations of model, compared to the standard model, on the reported outcomes is shown in Table 2. Broadly under each analysis the overall pattern of results comparing the three different estimates of need is similar. There were relatively large differences in the estimate of change in person-years lived with disease using the lifetable method (that allowed life expectancy to change) compared with comparative risk assessment method (that assumed life expectancy was unchanged). Assuming that physical activity did not affect colon cancer survival attenuated the estimated increase in person-years lived with colon cancer, such that uncertainty intervals included zero, and for breast cancer the point estimate changed from a small increase to a small decrease (uncertainty intervals including zero).

**Table 2:** Summary of model outcomes for different ‘structural’ configurations of the model for the ‘meeting guidelines’ scenario

	Standard model	‘Structural’ change to the standard model			
		PA has no effect on colon or breast cancer survival	PA effects incidence of other cancers	No lag between physical activity and change in disease risk	Only include walking, sport and recreational activity
*Increase in LE (days)*					
Women	**101 (75–131)**	**85 (60–115)**	**106 (79–138)**	**126 (92–170)**	**128 (95–164)**
Men	**89 (61–123)**	**82 (54–117)**	**95 (67–130)**	**103 (72–141)**	**115 (82–154)**
All	**95 (69–126)**	**84 (58–115)**	**101 (74–133)**	**115 (83–153)**	**115 (83–153)**
*Change in total incidence cases (%)*					
IHD	**−4.6 (−7.6 to −2.4)**	**−4.7 (−7.7 to −2.5)**	**−4.5 (−7.6 to −2.3)**	**−4.8 (−8.0 to −2.4)**	**−6.0 (−9.3 to −3.2)**
Stroke	**−4.6 (−7.9 to −2.1)**	**−4.7 (−8.1 to −2.2)**	**−4.5 (−7.8 to −1.9)**	**−4.8 (−8.3 to −2.0)**	**−6.0 (−9.7 to −2.8)**
Type 2 diabetes	**−2.5 (−4.4 to −1.1)**	**−2.6 (−4.4 to −1.1)**	**−2.5 (−4.3 to −1.1)**	**−2.8 (−4.7 to −1.2)**	**−4.0 (−6.6 to −1.9)**
Dementia	−0.4 (−3.6 to 1.9)	−0.7 (−3.9 to 1.6)	−0.2 (−3.4 to 2.1)	−2.2 (−7.2 to 1.2)	−0.8 (−4.9 to 2.0)
Breast cancer	−0.8 (−2.0 to 0.0)	−0.8 (−2.1 to 0.0)	−0.7 (−2.0 to 0.1)	**−1.6 (−3.3 to −0.3)**	**−1.6 (−3.3 to −0.3)**
Colon cancer	−0.6 (−2.5 to 0.8)	−0.7 (−2.5 to 0.7)	−0.5 (−2.4 to 0.9)	−1.7 (−4.6 to 0.5)	−1.3 (−4.0 to 0.7)
Lung cancer	**1.5 (1.1 to 2.0)**	**1.1 (0.8 to 1.5)**	−0.7 (−1.8 to 0.3)	**1.9 (1.3 to 2.5)**	**1.9 (1.4 to 2.5)**
Prostate cancer	**1.3 (0.9 to 1.7)**	**1.4 (0.9 to 1.9)**	0.5 (−0.3 to 1.2)	**1.5 (1.1 to 2.1)**	**1.6 (1.2 to 2.1)**
Pancreatic cancer	**1.7 (1.2 to 2.2)**	**1.5 (1.0 to 2.1)**	−2.0 (−8.1 to 1.4)	**2.1 (1.5 to 2.7)**	**2.1 (1.6 to 2.8)**
*Change in person-years with disease (%)*					
IHD	−1.2 (−4.2 to 1.4)	−1.3 (−4.5 to 1.3)	−1.0 (−4.3 to 1.5)	−1.1 (−4.5 to 1.6)	−2.5 (−6.1 to 0.7)
Stroke	**−3.1 (−6.0 to −1.1)**	**−3.2 (−6.1 to −1.2)**	**−3.0 (−5.9 to −1.0)**	**−3.1 (−6.3 to −1.0)**	**−4.7 (−8.1 to −2.0)**
Type 2 diabetes	**−1.5 (−3.2 to −0.2)**	**−1.6 (−3.3 to −0.3)**	**−1.4 (−3.1 to −0.1)**	**−1.5 (−3.3 to −0.1)**	**−2.8 (−5.2 to −0.8)**
Dementia	−0.6 (−3.7 to 1.6)	−0.9 (−4.1 to 1.3)	−0.4 (−3.6 to 1.8)	−2.5 (−7.4 to 0.9)	**−1.3 (−5.5 to 1.6)**
Breast cancer	0.8 (−0.50 to 1.7)	−0.1 (−1.3 to 0.6)	0.8 (−0.4 to 1.8)	0.5 (−1.2 to 1.8)	0.3 (−1.5 to 1.8)
Colon cancer	**4.4 (1.1 to 10.3)**	0.3 (−1.4 to 1.6)	**4.6 (1.1 to 10.4)**	4.1 (−0.1 to 10.2)	**4.9 (0.4 to 11.4)**
Lung cancer	**1.8 (1.3 to 2.4)**	**1.6 (1.1 to 2.2)**	−0.3 (−1.4 to 0.7)	**2.2 (1.5 to 3.0)**	**2.3 (1.7 to 3.0)**
Prostate cancer	**1.9 (1.3 to 2.6)**	**1.7 (1.2 to 2.5)**	**1.3 (0.5 to 2.2)**	**2.2 (1.6 to 2.9)**	**2.4 (1.7 to 3.2)**
Pancreatic cancer	**1.5 (1.1 to 2.0)**	**1.4 (0.9 to 1.9)**	−1.9 (−7.7 to 1.3)	**1.8 (1.3 to 2.4)**	**1.9 (1.4 to 2.5)**
*Change in person-years with disease (life expectancy unchanged) (%)*					
IHD	**−5.1 (−8.2 to −2.9)**	**−5.1 (−8.2 to −2.9)**	**−5.1 (−8.2 to −2.9)**	**−5.1 (−8.2 to −2.9)**	**−6.8 (−10.4 to −4.0)**
Stroke	**−5.2 (−8.3 to −2.9)**	**−5.2 (−8.3 to −2.9)**	**−5.2 (−8.3 to −2.9)**	**−5.2 (−8.3 to −2.9)**	**−6.9 (−10.4 to −4.0)**
Type 2 diabetes	**−3.8 (−6.1 to −1.8)**	**−3.8 (−6.1 to −1.8)**	**−3.8 (−6.1 to −1.8)**	**−3.8 (−6.1 to −1.8)**	**−5.1 (−8.0 to −2.5)**
Dementia	**−4.0 (−7.5 to −1.6)**	**−4.0 (−7.5 to −1.6)**	**−4.0 (−7.5 to −1.6)**	**−4.0 (−7.5 to −1.6)**	**−5.3 (−9.9 to −2.2)**
Breast cancer	**−2.4 (−4.0 to −1.2)**	**−2.4 (−4.0 to −1.2)**	**−2.4 (−4.0 to −1.2)**	**−2.4 (−4.0 to −1.2)**	**−3.2 (−5.2 to −1.7)**
Colon cancer	**−2.2 (−4.2 to −0.8)**	**−2.2 (−4.2 to −0.8)**	**−2.2 (−4.2 to −0.8)**	**−2.2 (−4.2 to −0.8)**	**−3.1 (−5.7 to −1.1)**
Lung cancer	0 (0 to 0)	0 (0 to 0)	−2.5 (−3.7 to −1.6)	0 (0 to 0)	0 (0 to 0)
Prostate cancer	0 (0 to 0)	0 (0 to 0)	−1.0 (−0.4 to −2.0)	0 (0 to 0)	0 (0 to 0)
Pancreatic cancer	0 (0 to 0)	0 (0 to 0)	−3.9 (−0.5 to −10.0)	0 (0 to 0)	0 (0 to 0)

Meeting guidelines scenario assumes that all adults who are not presently doing 5.75 marginal MET-hours of physical activity (equivalent to 150 min of walking per week) increase their physical activity to 5.75 marginal MET-hours, the physical activity level of adults who are doing more than 5.75 marginal MET-hours per week is unchanged; *IHD* ischaemic heart disease; bold type indicates that the uncertainty intervals do not include zero; Outcomes for lung cancer, prostate cancer and pancreatic cancer are included under all variants of the model for comparison. Physical activity only affects the incidence of lung cancer, prostate cancer or pancreatic cancer in the third model described as ‘PA effects incidence of other cancers’. In all other models physical activity does not affect the incidence of lung cancer, prostate cancer or pancreatic cancer

## Discussion

### Summary of main findings

Increases in physical activity were associated with a reduction in disease incidence and an increase in life expectancy. Generally, increases in physical activity were associated with a reduction in measures of need for healthcare (both incident cases and person-years lived with disease) over the life of the cohort. However, estimates of the effect of physical activity on indices of need, using a lifetable method that made allowance for change in survival, were more conservative than similar estimates made using comparative risk assessment methods (e.g. dementia, ischaemic heart disease) that did not make allowance for changes in survival. For some diseases, for which physical activity is protective, increases in physical activity might be associated with an increase in the person-years lived with disease (e.g. colon cancer).

### Strengths and limitations

The strengths of this study include: the explicit modelling of aging, modelling the effect of physical activity on mortality through a set of diseases, considering indices of healthcare need, long period of follow-up, and making allowance for a lag between physical activity and its effect on disease risk. We have also drawn comparisons between modelling techniques (lifetable vs. comparative risk assessment) to demonstrate the additional impact of modelling increased survival on the reported outcomes.

As with all modelling work a number of assumptions have been made. Some of the uncertainty associated with these assumptions has been explored by uncertainty and sensitivity analyses. While parametric and structural uncertainty affected the magnitude of the results, it did not affect the pattern of results comparing the different measures of need.

We have focused on the diseases for which physical activity is protective. The effect of increases in physical activity (and resultant increases in life expectancy) on other diseases whose incidence is age-dependent and independent of physical activity (e.g. some cancers) will be different. For such diseases increases in physical activity are likely to be associated with an increase in the both the number of incident cases and the person-years lived with disease (see pancreatic, lung and prostate cancers in Table 2 under the ‘standard model’).

We have modelled cancer as a chronic disease without recovery or remission. While this may not reflect the course of some cancers (i.e. remission or cure), it does reflect the convention of measuring cancer prevalence and the increasing recognition that cancer can be a chronic disease [65, 66]. We have considered only some measures of need for healthcare and have not considered severity, co-morbid illness, or costs, which could give a fuller picture of the impact on health and social care. It seems likely for some diseases (e.g. ischaemic heart disease, type 2 diabetes) that increases in physical activity will be associated with reductions in disease severity or improvements in quality of life [54, 67, 68], which the outcome measures do not reflect. This may be an important ‘health gain’, which we have not explicitly considered and is likely to have implications for healthcare utilisation.

We should be particularly cautious about the interpretation of data amongst the very old (aged 80 years and over). First, there is relatively limited data on disease parameters (incidence and prevalence) beyond age 90 years, and while mortality data is complete to 100 years, the coding of deaths in older age may be less reliable [69, 70]. Second, we have assumed that the effect of physical activity on disease incidence is similar (on a relative scale) throughout life, although its effect is much less studied in older age. Third, the increases in physical activity modelled in later life may be less achievable, either because of co-morbidities or limited cardiovascular reserve. Fourth, co-morbidities are more common in older age, and the effect of physical activity on disease risk when there are co-morbidities is not explicitly represented in a proportional life-table model.

Finally, we suggest our results should not read as forecasts as to what would happen from increases in physical activity in the future, in England. Changes in disease incidence or other risk factors (e.g. cardiovascular incidence has declined and life expectancy increased in the past 50 years) [71, 72] would affect such forecasts and have not been considered. Rather one should see the work as an exploration of the effect of increases in physical activity assuming that other factors are unchanged.

### Model validity: comparisons with other estimates

Comparing some outputs of our model, with other published estimates may serve as a form of model validation. Our estimate of the increase in life expectancy (95 days) attributed to ‘meeting guidelines’ is less than a recent comparable estimate (256 days) if everyone in the UK walked briskly for at least 20 min daily [2]. It is also less than an estimate of the increase in life expectancy from everyone aged between 40 and 65 years of age meeting physical activity guidelines (168 days), using a modelling approach that shared some characteristics with ours [73]. Both of these studies modelled the effect of physical activity on mortality directly, rather than through disease states as we did. Other methodological differences may explain the discrepancies (e.g. how ‘inactivity’ equates to marginal MET-hours).

We can also draw comparisons with estimates of the effect of physical activity on measures of need made using comparative risk assessment methods. Generally such estimates tend to suggest a bigger effect of physical activity than we observed [8, 26, 27, 29]. For example modest increases in walking and cycling were estimated to reduce incident cases for the diseases we consider here by 5% (for colorectal cancer) to 11.5% (for type 2 diabetes) [26]. Different model parameters and differences in the scenarios may explain the differences.

Taken together these findings may suggest that our model is under estimating the effect of physical activity on disease, relative to other models. However our conclusions primarily relate to the pattern of results, which the sensitivity analyses suggest are largely unaffected by changing the dose of (and thus the effective efficacy of) physical activity, rather than absolute estimates.

### Effect of physical activity on need: comparison with other work

Limited other work has explored the effect of changes in physical activity on specific diseases. Past work has also tended to frame findings around average changes for an individual (e.g. disease expansion and compression) [20, 22], although such measures can be compared to our measure of person-years with disease (See supplementary material).

Previous work has reported that increases in physical activity from none or low levels to moderate or high levels were associated with a reduction in the average number of years lived with disability [19]. Whilst we have not estimated all-cause morbidity we note that the general trend was for the person-years lived with disease to decrease.

Two modelling studies reported that increases in physical activity (during mid-life) were associated with small non-significant increases in average years lived with cardiovascular disease [19, 20], and a third reported a significant decrease in average years lived with dementia [74]. While the central estimates are discordant (we found small non-significant decrease for ischaemic heart disease and dementia), the uncertainty intervals overlap. Lifetable modelling has also been used to describe the effect of other risk factors on years lived with cardiovascular disease [21–23]. Smoking cessation was associated with an increase in the average number of years lived with cardiovascular disease (equivalent to an increase in the person-years lived with disease) [22]. In contrast reductions in body weight were associated with a reduction in the average number of years lived with cardiovascular disease [22, 23]. These findings are consistent with our general observation that an ‘improvement’ in a risk factor can be associated with either an increase or a decrease in person-years lived with disease, which may not be readily predicted from measures of relative risk alone.

We are not aware of any studies directly comparing lifetable methods with comparative risk assessment methods, nor any studies comparing health impact modelling that makes allowance for changes in life expectancy with methods that do not.

### Interpretation

The effect of physical activity on the healthcare need relates to disease epidemiology and the three effects we outlined in the introduction (see Fig. 1). The effect varies for different diseases.

Type 2 diabetes and stroke show a similar pattern (decrease in incident cases, decrease in person-years lived with disease, and both these estimates are not too discordant from estimates made using comparative risk assessment methods). For these diseases the incidence effect is dominant. This reflects a relatively strong effect of physical activity on relative risk of incidence and the absence of a disease survival effect (i.e. physical activity does not affect disease case fatality). For type 2 diabetes, the fall in incidence rate with age also suggests that population aging is less important.

Dementia is different (small decreases in incident cases and person-years lived with disease that are close to zero and much less than estimates made using comparative risk assessment methods). The incidence of dementia increases sharply with age, such that the population aging effect is important. While a few cases of dementia were prevented, more commonly the onset of dementia was postponed.

Ischaemic heart is different again (large decrease in incident cases but relatively small decrease in person-years lived with disease). The disease survival effect is important, whilst cases of disease are prevented those with disease are living longer.

For colon and breast cancer the disease survival effect is also important. In addition few cases of colon and breast cancer are prevented, which may be attributed to population aging and a rise incidence with age and/or a relatively weak effect of physical activity on incidence. For colon cancer the combination of these effects meant that increases in physical activity were associated with a relatively large increase in person-years with colon cancer. The large magnitude of the increase is, in large part, attributable to a strong effect of physical activity on survival after diagnosis (see Table 2). However given that this estimate is based only on observational studies, which may be subject to confounding by indication (see Uncertainty and Sensitivity Analyses in the Methods), the large increase in person-years lived with colon cancer should be interpreted cautiously. Moreover, given that, within the model, survival with breast or colon cancer would include many people without ongoing symptoms, the clinical importance of an increase in person-years lived with breast or colon cancer for the health service (and individuals) is likely to be less than for other diseases (e.g. dementia).

For some diseases, increases in physical activity were associated with decreases in the mean age of onset. Whilst this may appear counter-intuitive, particularly given that the rightward shift of the disease curve (Fig. 6) suggesting later onset, one should remember that the estimates reflect the mean age *for those who develop* disease. Thus, it is possible for the mean age of onset to increase, whilst the age of onset of those who develop disease is delayed if cases of disease are prevented predominantly in those who would have developed the disease at old age.

### Implications

Broadly our work suggests that changes in life expectancy are important when evaluating or formally estimating the effect of physical activity on indices of need for healthcare.

Whilst we have only considered physical activity, in the context of a single setting (England), we think our broad conclusion, concerning the importance of considering changes in life expectancy, is likely to extend to other risk factors and other settings. An increase in disease incidence with age and the three different effects are common to other risk factors and diseases. Whilst the nature and strength of the association between other risk factors and diseases may differ, other important risk factors for non-communicable diseases (e.g. smoking, alcohol and diet) are all associated with both mortality and disease incidence.

The work has two important implications. First it suggests that public health officials and policy makers should be more cautious about claiming that interventions designed to reduce risk will lead to large reductions in need for healthcare, with consequent reductions in utilisation of healthcare. Whilst such resource-based arguments may be a popular way to frame arguments [7, 9] and may sometimes be appropriate, they should be tempered with realism. Instead it may be more appropriate to frame arguments around improvements in health.

Similarly it is common to talk of “prevention”, but our results suggest that risk reduction may result in little or no prevention of some diseases. The term “prevent” may be sometimes be appropriate (e.g. the effect of physical activity on diabetes), but sometimes “delay” may be most appropriate (e.g. the effect of physical activity on dementia). A sensible phrase may be “risk reduction which may delay or prevent disease onset”, reflecting the language in some recent publications [75, 76].

The second important implication concerns public health modelling. Researchers who undertake such modelling should consider using lifetable models or other tools to make allowance for increased life expectancy and the delay in onset of the disease. Much of the work that considers the benefits of physical activity (or costs of physical inactivity) and other behaviours uses comparative risk assessment modelling [8, 26, 29, 77].

Our paper also suggests grounds for caution when making another common assumption that an aging population leads to increased need for health and social care [13, 78]. For example there have been forecasts that population aging will lead to a significant rise in need for dementia care [79, 80]. Our work suggests that if changes occur in a risk factor, which is a risk factor for both mortality and for disease incidence, then it is possible for the population to age whilst the need for healthcare (at least for some diseases) is relatively unchanged. We note that recent research suggests that the number of people living with dementia is largely unchanged in the last 10–20 years, despite population aging [11, 81].

Finally, despite a note of caution about implications for healthcare utilisation, our work does underscores the benefits of physical activity for health (e.g. increased life expectancy and prevention of cases or delay in disease onset). For most diseases, even making allowance for changes in life expectancy, measures of need tend to decreases.

### Future research

This work only partially answers the question about the extent to which increases in physical activity, when considering its effect on survival, affect the actual need for healthcare. Future work could explore the effect on all-cause disability, considering disease severity and other diseases (including those whose incidence increases with age but is independent of physical activity). It would also be informative to describe the impact on a population of mixed ages (rather than a birth cohort) over a time horizon that is more prescient for decision makers (e.g. 5–20 years), and to explore the impact on changes in physical activity restricted to particular phases of life (e.g. mid-life). To understand the economic implications a full economic appraisal would be required. This could consider other factors (e.g. deferment of cost if disease is delayed) and other sources of economic costs or benefits (e.g. tax base from an increased population, productivity of a working age population that is healthier, increased pension costs from an older population).

Further work should also seek to understand the limits of life-table models, and the extent to which violations of the underlying assumptions around disease independence affect the model outcomes. It would also be of value to repeat this work with other risk factors, notably smoking which has a pronounced effect on mortality [82].

## Conclusions

Our work reaffirms the benefits of physical activity for health (increased life expectancy and prevention of or delay in disease occurrence). For most diseases for which physical activity is protective, increases in physical activity are associated with decreases in healthcare need. However incident cases of disease may be delayed or the period of time lived with disease may increase, such that the decreases in need may be relatively small and less than is sometimes expected.

We suggest some areas of public health practice should be more cognisant of the effect of increased survival on indices of need for healthcare. Public health officials should consider exercising greater caution when making claims about whether and the extent to which increases in physical activity or improvement in other risk factors will reduce need for health or social care. Instead the benefits of risk reduction interventions may be better described in terms of improved health (preventing or delaying disability and delaying death). Public health modellers should consider the potential impact of changes in longevity when designing health impact models.

## Electronic supplementary material

Below is the link to the electronic supplementary material.

Supplementary material 1 (DOCX 22 kb)

Supplementary material 2 (DOCX 141 kb)

Supplementary material 3 (DOCX 267 kb)
