# Supplementary material for: The modelled impact of increases in physical activity: the effect of both increased survival and reduced incidence of disease
Source: Eur J Epidemiol. 2017 Mar 3;32(3):235–50. doi: 10.1007/s10654-017-0235-1 (PMC5380706; doi:10.1007/s10654-017-0235-1)
Supplement: Supplementary file 1 — Supplementary material 1 (DOCX 22 kb) [file 10654_2017_235_MOESM1_ESM.docx]

**Equating average number of years lived with disease with person-years lived with disease**

A common metric in previous modelling studies is estimating the mean number of years an individual lives with diseases.[1,2] This measure may be estimated by dividing the total number of person-years lived with disease by the total population.

In our study the population size was fixed at 100,000 in all scenarios. Thus increases in the person-years lived with disease will result in an increase in the average number of years lived with disease. Similarly decreases in the person-years lived with disease will result in a decrease in the average number of years lived with disease.

Similarly disease expansion (an absolute increase in the average number of years lived with disease) is equivalent to an increase in the person-years lived with disease and disease compression (an absolute decrease in the average years lived with disease) is equivalent to a decrease in person years lived with disease.

**References**

1. Nusselder WJ, Franco OH, Peeters A, Mackenbach JP. Living healthier for longer: comparative effects of three heart-healthy behaviors on life expectancy with and without cardiovascular disease. BMC Public Health. 2009;9: 487. doi:10.1186/1471-2458-9-487

2. Nusselder WJ. Smoking and the compression of morbidity. J Epidemiol Community Heal. 2000;54: 566–574. doi:10.1136/jech.54.8.566
