# Supplementary material for: The modelled impact of increases in physical activity: the effect of both increased survival and reduced incidence of disease
Source: Eur J Epidemiol. 2017 Mar 3;32(3):235–50. doi: 10.1007/s10654-017-0235-1 (PMC5380706; doi:10.1007/s10654-017-0235-1)
Supplement: Supplementary file 2 — Supplementary material 2 (DOCX 141 kb) [file 10654_2017_235_MOESM2_ESM.docx]

**Methods Appendix**

Calculation of marginal MET-hours

Physical activity levels, measured in marginal MET-hours, were estimated by summing the product of physical activity duration and intensity, following the approach used previously.[1]

Marginal MET-hours are similar to MET-hours, which discounts energy expenditure due to basal metabolism (as this energy expenditure would occur independent of physical activity).^105^ Using marginal-MET instead of MET results in low intensity activities undertaken for a long period of time ‘scoring’ less, and correspondingly high intensity activities undertaken for short periods of time score more. Marginal MET estimates for any activity are estimated by subtracting one from reported MET value for that given activity.

Estimates of the duration of each activity were made from the International Physical Activity Questionnaire (IPAQ) within the Health Survey for England. This contains information on the type, duration, frequency and sometimes intensity of all forms of physical activity undertaken in the past four weeks. Our analysis only considered forms of physical activity that were moderate or vigorous in nature, partly reflecting their emphasis in current guidance and partly because of the large empirical evidence base that considers moderate and vigorous physical activity, which we used to parametrise our model.[2–4]

Standard MET values for each type of physical activity were taken from Ainsworth’s Compendium of physical activity.[5] Based on the questions in the Health Survey of England we choose representative activities from the Ainsworth Compendium of Physical Activities 2011 to estimate the intensity of physical activity (measured in METS). Where there activity described in the IPAQ did not clearly map to an activity in the Ainsworth Compendium of Physical Activities, we identified a set of activities that could fit the description of the physical activity recorded by the RPAQ, and took an average (median) value. The values we used are shown in table A1.

The ‘dose’ of physical activity for each activity were estimated by taking the product of weekly duration of the activity (in hours) and the intensity of the activity (measured in marginal METS – i.e. MET value minus one). The total ‘dose’ of physical activity was then estimated by summing together the estimates for each activity. We included the following types of physical activity occupational (walking, stair climbing, lifting), domestic (housework and gardening), travel (walking) and recreational.

Estimates of the proportion of the population by age who are meeting guidelines is shown in Table A2 when all forms of physical activity (as listed above where included), and when only including walking and recreational activity.

Modelling the effect of physical activity level on disease

Most studies report a curvilinear between physical activity and disease or mortality (e.g. Figure A1), although different approaches have been used to mathematically describe the relationship between physical activity level and disease risk (e.g. log linear, square root transformation).[6–9] Following the approach used by others,[10] we assumed changes in risk of disease to be log linearly associated with a power transformation of the physical activity exposure, where the power transformation took a value of 0.5 (range 0.25 to 1.0 with a triangular distribution) for all relationships, following the range used within ITHIM.[1]

This relationship can be written as follows:

**RR = a^(PA dose/b)^t^**

Where RR=relative risk, PA dose=physical activity dose measured in marginal MET-hours, t=power transformation, a is the reported relative risk from the meta-analysis, b is the physical activity level at which the reported relative risk occurred.

Examples of this relationship are shown in Figure A1. Where possible I took estimates of relative risk and physical activity level (parameters a and b) from the original ITHIM model.[1] Where estimates of the these parameters were not given in the work of Woodcock et al,[1] we sought estimates from the literature using the approach outlined by Woodcock et al.[1]

First we sought to identify the most suitable recent meta-analysis that described the relationship between physical activity and relative risk. The primary estimate of effect (relative risk) from the meta-analysis was identified. The corresponding measure of physical activity dose at which this relative risk is likely to occur is much less commonly reported. We then sought to identify the largest single study of moderate to vigorous physical activity within the meta-analysis. We then estimated the median exposure in the highest exposure group of whichever group corresponds to physical activity level for the effect size used in the meta-analysis. The derivation of these values from key studies is set out in Table A3.

A summary of the parametric values used to model the effect of physical activity on disease incidence, disease survival and mortality is given in Table A4.

Literature search to describe the effect of physical activity on disease survival

We identified studies that described the effect of physical activity on disease survival using the following search terms in Pubmed on 1 April 2015: “systematic review” or “meta-analysis” and “physical activity” or “exercise” and “survival” or “case fatality” and “colon cancer” (including bowel cancer and colo-rectal cancer) or “breast cancer” or “ischaemic heart disease” (including myocardial infarction, IHD and cardiovascular disease) or “stroke” (including cerebrovascular accident) or “dementia” or “diabetes”.

We only modelled an effect on case fatality for ischaemic heart disease, colon cancer and breast cancer. There was insufficient evidence to justify an effect on mortality for stroke or dementia. For stroke, while physical activity is recommended after stroke, the nature of the activity has to be tailored to the individual and any residual disability, it is advocated primarily as a means to improve functional performance and quality of life, and we found no evidence (either observational epidemiology or randomised controlled trials) that quantified the relationship between physical activity after stroke and mortality due to stroke.[11, 12] For dementia, we found no studies (either observation or randomised controlled trials) that quantified the relationship between physical activity after a diagnosis of dementia and subsequent mortality from dementia. As others have done, we have not included a direct effect of physical activity on diabetes related survival, because much of the mortality attributable to diabetes occurs through ischaemic heart disease and stroke, and modelling diabetes in this way would likely violate the underlying assumptions of a proportional multi-state life table model.[13, 14]

Literature search to describe the effect of physical activity on incidence of pancreatic, prostate and lung cancer

A recent review article highlighted new evidence of associations between physical activity and risk of several other cancers.[15] For three important cancers (pancreatic, prostate and lung) that form the five main causes of cancer mortality along with breast and colon cancer in the UK, the evidence of a causal association was described as “probable” or “possible”.[15]

We identified studies that described the effect of physical activity on incidence of pancreatic, lung and prostate cancer using the following search terms in Pubmed on 1 April 2015: “systematic review” or “meta-analysis” and “physical activity” or “exercise” and “pancreatic cancer” or “lung cancer” or “prostate cancer”.

For lung cancer we assumed that physical activity only has an affect amongst smokers of lung cancer attributable to smoking, in keeping with recent findings.[16, 17] Given the attributable fraction for smoking with respect to lung cancer is 80% [18] we modelled an incidence of lung cancer that was 80% of that which we derived from routine statistics.

Modelling the lag between physical activity and disease

To model the lag between physical activity and the outcome of interest, we used population impact fraction that represented the physical activity level x years previously, where x is the lag being simulated for the outcome of interest. We then took the average of the population impact fractions over the range of time thought to be important. For example, if for IHD we assume a one to five year lag between physical activity and risk reduction for men aged 50 years. We would take an average of the population impact fraction based on the levels of physical activity undertaken by men aged 49 years, 48 years, 47 years, 46 years and 45 years.

There is very limited empirical evidence that directly addresses questions concerning the lag between physical activity and change in disease incidence or case fatality. However, the causal pathway between physical activity and cardio-metabolic disease, and its associated lags, are relatively well described. Randomised controlled trials suggest that physical activity can affect metabolic risk factors for ischaemic heart disease and stroke within a period of weeks.[19–22] The lag between changes in these risk factors and change in disease risk occurs within a period of years. For example statins (which effect serum cholesterol) effect cardiovascular risk within a year, with the full effect being observed as early as six years.[23] Trials of blood pressure lowering agents, which have been shown to reduce incidence of ischaemic heart disease, are in the period one to five years.[24] The prospective studies of the effect of physical activity on ischaemic heart disease and stroke range between two and 29 years, with a mean of 14 and 13 years respectively. Randomised controlled trials of patients with pre-diabetes that have shown an effect on diabetes risk have typically had follow-up period of around two to five years.[25, 26] Given this we have assumed a lag of one to five years between changes in physical activity and cardio-metabolic disease risk.

For cancer and physical activity there is no such similar evidence to inform estimates, although we note studies for smoking and lung cancer suggest a lag period of 25-35 years.[27–29] For physical activity and colon cancer the follow-up in the prospective studies was four to 24 years,[30] and for breast cancer it was six to 28 years.[31] This might suggest lag periods for physical activity and cancer may not be as long as 25-35 years. Given this large uncertainty we have assumed a lag of one to 30 years between changes in physical activity and cancer.

We have also assumed a similar lag (one to 30 years) for dementia, reflecting the long aetiology period, although we note that dementia may share some causal pathways with cardio-metabolic disease.[32–34]

We assumed a shorter lag of the effect of physical activity on survival from cancer reflecting the duration of the trials that have shown an association between physical activity and survival from breast (follow-up three to six years) and colon cancer (follow-up five to 12 years).[35]

Values used to model the lags are shown in Table A4.

Estimating transition hazards using DISMOD

DISMOD II was originally developed for the Global Burden of Disease project to estimate epidemiological parameters were the necessary data for estimating burden of disease is not present. For example it can be used to estimate incidence from data on prevalence and mortality. We also used DISMOD to provide estimates of incidence and case fatality for each one year age group, where these parameters were only given for five- or ten-year age bands. A variety of epidemiological parameters can be used as inputs for DIMOD II. In practice we only used the following: incidence, prevalence, mortality and relative risk of mortality.

The data used as inputs for DISMOD were taken from a variety of sources and is set out in Table A4. We identified and choose these datasets based on our knowledge of the strengths and weaknesses of the different datasets and where appropriate respecting the following hierarchy: routine and comprehensive datasets (England or UK), representative national surveys, single studies conducted in England or the UK. We followed the following procedure in DISMOD to estimate disease incidence and case fatality. First the raw data (often by five or ten year age bands) from two epidemiological parameters was entered into DISMOD II. This data was then smoothed and where possible modelled as a mathematical function (or best fit curve). In the second stage the adjusted inputs were used to estimate a new set of epidemiological parameters for each one year age increment. Both sets of epidemiological parameters used as inputs were weighted equally unless there was a strong a priori reason why one data source was considered more reliable than the other.

Calculation of Potential Impact Fractions

Relative risks were calculated under the baseline scenario (no change in physical activity) and under the alternative or counterfactual scenario (an increase in physical activity levels). The potential impact fraction is then estimated as the weighted sum of the ratio of the change in relative risk to the absolute relative risk at baseline, defined as:


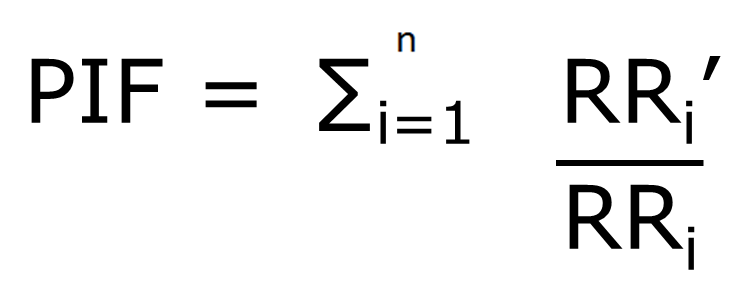


Where PIF is the Potential Impact Fraction, RR_i_’ is the relative risk under the counterfactual scenario and RR_i_ is the relative risk under the baseline scenario. This is equivalent to estimating a Population Attributable Fraction (PAF) as defined in comparative risk assessment models and Global Burden of Disease modelling.[36] Although PAF values are based on eliminating a known risk factor or shifting the population to an ‘idealised exposure’, in contrast the PIF values are based on the effect of changing physical activity levels as described in the relevant scenario.[1]

To reflect the different baseline levels of physical activity by age and sex, population impact fractions were calculated for age (16-19 years, 20-29 years, and then in 10 year blocks until 90-100 years) and sex strata, rather than for the population as a whole.

**Table A1: Summary of values extracted from the Ainsworth Compendium of Physical Activity and used to estimate marginal MET-hours**

| **Activity described in Health Survey for England** | **Physical Activity Intensity (METS)** | **Source and rationale** |
| --- | --- | --- |
| **Occupation** |  |  |
| Occupation, Walking at work | 3.5 | Median value of the following codes: walking on the job at speeds of less than 2mph to 3.5mph (11791, 2.0 METS; 11792, 3.5 METS; 11793, 4.3 METS), and walking on the job carrying light objects (11795, 3.5 METS). |
| Occupation, Climbing stairs/ladders | 6.3 | Median value of the following codes: carrying light load upstairs (17026, 5.0 METS), stair climbing or climbing a ladder (17130, 8.0 METS), stair climbing at a slow pace (17133, 4.0 METS), stair climbing at a fast pace (17134, 8.8 METS), climbing hills with no load (17033, 6.3 METS). |
| Occupation, Lifting, carrying or moving heavy loads | 7.5 | Median of walking or standing whilst carrying objects weighing from 25lbs to 100lbs or more (11820, 5.0 METS; 11830, 6.5 METS; 11840, 7.5 METS; 11850, 8.5 METS), loading and unloading truck (11766, 6.5 METS), standing and continuously lifting items (11615, 4.5 METS), carrying heavy loads (11050, 8.0 METS), moving boxes (11060, 8.0 METS), and moving or carrying objects of 75lbs or more (11490, 7.5 METS) |
| **Domestic** |  |  |
| Heavy housework | 3.5 | Median of multiple household tasks at light (05025, 2.8 METS), moderate (05026, 3.5 METS) and vigorous intensity (05027, 4.3 METS) |
| Heavy manual work at home (DIY, gardening or building work) | 4.15 | Median of gardening – general (08245, 3.8 METS), and home repair at light (06126, 2.5 METS), moderate (06127, 4.5 METS) and vigorous intensity (06128, 6.0 METS). |
| Walking at a brisk or fast pace (4mph or greater) | 3.9 | Median of walking on a flat surface from 2.5mph to 4mph (17170, 3.0 METS; 17190, 3.5 METS; 17200, 4.3 METS; 17220, 5.0 METS) |
| **Sport** |  |  |
| Swimming | 6.0 | Swimming general (18310) |
| Cycling | 6.8 | Bicycling to/from work, self-selected pace (01015) |
| Working out (e.g. weight training or exercise bike) | 6.0 | Median of exercise bike (02010, 7.0 METS), weight lifting (02030, 6.0 METS), and calisthenics (02020, 3.8 METS) |
| Aerobics, keep fit and gymnastics | 7.3 | Aerobics (03015, 7.3 METS) – assume this is predominantly aerobics/keep fit rather than gymnastics |
| Dancing | 5.0 | Ballet, modern, jazz or general dancing (03010) |
| Running or jogging | 8.4 | Median of jogging in general (12020, 7.0 METS) and running at 6mph (12050, 9.8 METS) |
| Football or rugby | 7.65 | Median of soccer – competitive (15605, 10.0 METS), soccer – casual (15610, 7.0 METS), rugby – competitive (15560, 8.3 METS), and rugby – non-competitive (15562, 6.3 METS) |
| Badminton or tennis | 7.0 | Median of tennis – general (15675, 7.3 METS), badminton – social (15030, 5.5 METS) and badminton – competitive (15020, 7.0 METS) |
| Squash | 7.3 | Squash general (15652) |
| Exercises (e.g. press ups) | 8.0 | Calisthenics (02020) |
| Any other sport | 6.0 |  |
| **Travel** |  |  |
| Walking | Dependent on speed | Slow = 2.5 METS (17152) was discounted unless the participant was aged over 65 years and reported breathlessness on walking, in which case we assumed this was equivalent to achieving moderate intensity physical activity; average pace = 3.0 METS (17170); fairly brisk = 3mph = moderate pace = 3.3 METS (17190); fast pace = 4.0mph = 5.0 METS (17220) |

**Table A2: Proportion of population meeting physical activity guidelines within the model**

|  | **Percentage meeting guidelines (%)** | |
| --- | --- | --- |
|  | **Broad definition** | **Narrow definition** |
| **Women** |  |  |
| 16-29 years | 35.6% | 13.6% |
| 30-39 years | 40.4% | 12.8% |
| 40-49 years | 42.3% | 10.4% |
| 50-59 years | 43.7% | 9.2% |
| 60-69 years | 27.0% | 9.6% |
| 70-79 years | 11.5% | 5.1% |
| 80 years and over | 2.7% | 1.1% |
| **Men** |  |  |
| 16-29 years | 55.5% | 33.6% |
| 30-39 years | 55.5% | 20.4% |
| 40-49 years | 51.6% | 13.6% |
| 50-59 years | 47.3% | 14.1% |
| 60-69 years | 34.3% | 12.9% |
| 70-79 years | 20.9% | 10.4% |
| 80 years and over | 4.1% | 1.4% |

Broad definition includes occupational activity (walking, stair climbing, lifting), domestic (housework and gardening), travel activity (walking) and recreational activity; the narrow definition only includes physical activity due to walking, sport or other recreational activities

**Table A3: Summary of parameters characterising the relationship between physical activity and risk**

| Disease | Study | Relative Risk (95% CI) | Physical activity level (marginal MET-hours per week) | Standardised  Relative Risk | Lag (years) |
| --- | --- | --- | --- | --- | --- |
| Incident Disease |  |  |  |  |  |
| Breast cancer | Monninkhof et al, 2007[31] | 0.94 (0.92-0.97) | 3.5 | 0.97 | 1-30 |
| Cardiovascular disease | Hamer et al, 2008[37] | 0.84 (0.79-0.90) | 5.4 | 0.94 | 1-5 |
| Colon Cancer | Harriss et al, 2009[30] | Men: 0.80 (0.67-0.96)  Women: 0.86 (0.76-0.98) | Men: 23  Women: 14 | 0.98 | 1-30 |
| Diabetes | Jeon et al, 2006[38] | 0.83 (0.75-0.91) | 10 | 0.94 | 1-5 |
| Dementia | Hamer et al, 2009[39] | 0.72 (0.60-0.86) | 24.5 | 0.95 | 1-30 |
| Lung cancer | Buffart et al,  2014[17] | 0.82 (0.77-0.87) | 21 | 0.97 | 1-30 |
| Pancreatic cancer | O’Rorke et al,  2010[40] | 0.72 (0.52-0.99) | 24 | 0.95 | 1-30 |
| Prostate cancer | Liu et al,  2011[41] | 0.90 (0.84-0.95) | 28 | 0.98 | 1-30 |
| Case Fatality |  |  |  |  |  |
| Breast cancer | Schmid et al, 2014[35] | 0.72 (0.60-0.85) | 24 | 0.94 | 1-5 |
| Colon cancer | Schmid et al, 2014[35] | 0.61(0.40-0.92) | 11 | 0.89 | 1-5 |
| Ischaemic Heart Disease | Heran et al,  2011 [42] | 0.87 (0.75-0.99) | 6 | 0.90 | 1-5 |

Standardised relative risk is the relative risk re-calculated for an increase of one marginal MET-hour per week. This table is based on work by Woodcock et al, 2009 and has subsequently been extended.

**Table A4: Sources of disease parameters used as inputs for DISMOD to estimate transition hazards for disease models**

| Disease | First Parameter | | Second Parameter | |
| --- | --- | --- | --- | --- |
|  | Parameter | Source | Parameter | Source |
| Breast cancer | Mortality | Mortality Statistics 2011[43] | Incidence | National Cancer Registry (2011)[44] |
| Colon Cancer | Mortality | Mortality Statistics 2011[43] | Incidence | National Cancer Registry (2011)[44] |
| Lung cancer | Mortality | Mortality Statistics 2011[43] | Incidence | National Cancer Registry (2011)[44] |
| Pancreatic cancer | Mortality | Mortality Statistics 2011[43] | Incidence | National Cancer Registry (2011)[44] |
| Prostate cancer | Mortality | Mortality Statistics 2011[43] | Incidence | National Cancer Registry (2011)[44] |
| Ischaemic Heart Disease | Mortality | Mortality Statistics 2012[45] | Prevalence | Health Survey for England 2012[46] |
| Stroke | Mortality | Mortality Statistics 2012[45] | Prevalence | Health Survey for England 2012[46] |
| Diabetes | Standardised mortality rate | National audit of general practice 2011-12[47] | Prevalence | National audit of general practice 2011-12[47] |
| Dementia | Relative risk of mortality | Analysis of primary care data[48] | Incidence | CFAS II study[33] |

**Figure A1: Diagram showing a hypothetical relationship between physical activity level and relative risk using different power transformations**


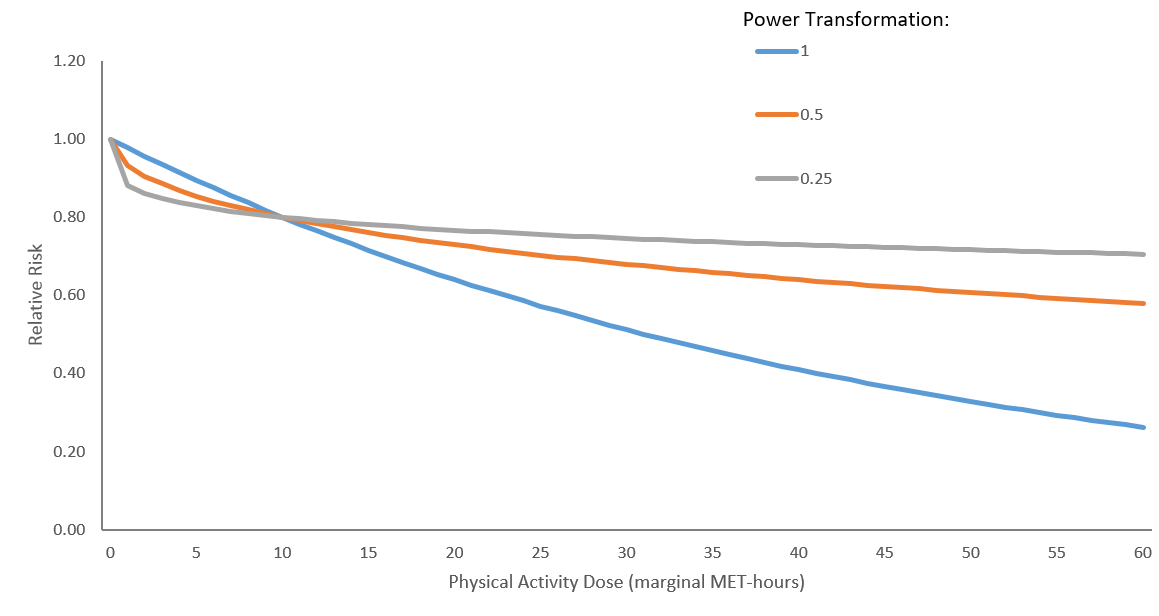


In this example, a=0.80, and b=10 marginal MET-hours

**References for methods**

1. Woodcock J, Edwards P, Tonne C, Armstrong BG, Ashiru O, Banister D, Beevers S, Chalabi Z, Chowdhury Z, Cohen A, Franco OH, Haines A, Hickman R, Lindsay G, Mittal I, Mohan D, Tiwari G, Woodward A, Roberts I: **Public health benefits of strategies to reduce greenhouse-gas emissions: urban land transport**. *Lancet* 2009, **374**:1930–1943.

2. Bull F, the Expert Working Groups: *Technical Report. Physical Activity Guidelines in the UK: Review and Recomendations*. Loughborough; 2010.

3. Haskell WL, Lee IM, Pate RR, Powell KE, Blair SN, Franklin BA, Macera CA, Heath GW, Thompson PD, Bauman A: **Physical activity and public health: updated recommendation for adults from the American College of Sports Medicine and the American Heart Association**. *Circulation* 2007, **116**:1081–1093.

4. Warburton DE, Charlesworth S, Ivey A, Nettlefold L, Bredin SS: **A systematic review of the evidence for Canada’s Physical Activity Guidelines for Adults.** *Int J Behav Nutr Phys Act* 2010, **7**:39.

5. Ainsworth BE, Haskell WL, Herrmann SD, Meckes N, Bassett DR, Tudor-Locke C, Greer JL, Vezina J, Whitt-Glover MC, Leon AS: **2011 Compendium of Physical Activities: a second update of codes and MET values.** *Med Sci Sports Exerc* 2011, **43**:1575–81.

6. Woodcock J, Franco OH, Orsini N, Roberts I: **Non-vigorous physical activity and all-cause mortality: systematic review and meta-analysis of cohort studies.** *Int J Epidemiol* 2011, **40**:121–38.

7. Kyu HH, Bachman VF, Alexander LT, Mumford JE, Afshin A, Estep K, Veerman JL, Delwiche K, Iannarone ML, Moyer ML, Cercy K, Vos T, Murray CJL, Forouzanfar MH: **Physical activity and risk of breast cancer, colon cancer, diabetes, ischemic heart disease, and ischemic stroke events: systematic review and dose-response meta-analysis for the Global Burden of Disease Study 2013.** *BMJ* 2016, **354**:i3857.

8. Kelly P, Kahlmeier S, Götschi T, Orsini N, Richards J, Roberts N, Scarborough P, Foster C: **Systematic review and meta-analysis of reduction in all-cause mortality from walking and cycling and shape of dose response relationship.** *Int J Behav Nutr Phys Act* 2014, **11**:132.

9. Wahid A, Manek N, Nichols M, Kelly P, Foster C, Webster P, Kaur A, Friedemann Smith C, Wilkins E, Rayner M, Roberts N, Scarborough P: **Quantifying the Association Between Physical Activity and Cardiovascular Disease and Diabetes: A Systematic Review and Meta-Analysis.** *J Am Heart Assoc* 2016, **5**:e002495.

10. Woodcock J, Tainio M, Cheshire J, O’Brien O, Goodman A: **Health effects of the London bicycle sharing system: health impact modelling study.** *BMJ* 2014, **348**(feb13_1):g425.

11. Boysen G, Krarup L-H, Zeng X, Oskedra A, Kõrv J, Andersen G, Gluud C, Pedersen A, Lindahl M, Hansen L, Winkel P, Truelsen T: **ExStroke Pilot Trial of the effect of repeated instructions to improve physical activity after ischaemic stroke: a multinational randomised controlled clinical trial.** *BMJ* 2009, **339**(jul20_3):b2810.

12. Billinger SA, Arena R, Bernhardt J, Eng JJ, Franklin BA, Johnson CM, MacKay-Lyons M, Macko RF, Mead GE, Roth EJ, Shaughnessy M, Tang A: **Physical Activity and Exercise Recommendations for Stroke Survivors: A Statement for Healthcare Professionals From the American Heart Association/American Stroke Association.** *Stroke* 2014, **45**:2532–2553.

13. Barendregt JJ, Van Oortmarssen GJ, Van Hout B a, Van Den Bosch JM, Bonneux L: **Coping with multiple morbidity in a life table.** *Math Popul Stud* 1998, **7**:29–49, 109.

14. Cobiac LJ, Vos T, Barendregt JJ: **Cost-effectiveness of interventions to promote physical activity: a modelling study.** *PLoS Med* 2009, **6**:e1000110.

15. Clague J, Bernstein L: **Physical activity and cancer.** *Curr Oncol Rep* 2012, **14**:550–8.

16. Zhong S, Ma T, Chen L, Chen W, Lv M, Zhang X, Zhao J: **Physical Activity and Risk of Lung Cancer: A Meta-analysis.** *Clin J Sport Med* 2015.

17. Buffart LM, Singh AS, van Loon ECP, Vermeulen HI, Brug J, Chinapaw MJM: **Physical activity and the risk of developing lung cancer among smokers: a meta-analysis.** *J Sci Med Sport* 2014, **17**:67–71.

18. Parkin DM: **2. Tobacco-attributable cancer burden in the UK in 2010.** *Br J Cancer* 2011, **105 Suppl**:S6–S13.

19. Kessler HS, Sisson SB, Short KR: **The potential for high-intensity interval training to reduce cardiometabolic disease risk.** *Sports Med* 2012, **42**:489–509.

20. Cesa CC, Sbruzzi G, Ribeiro RA, Barbiero SM, de Oliveira Petkowicz R, Eibel B, Machado NB, Marques R das V, Tortato G, dos Santos TJ, Leiria C, Schaan BD, Pellanda LC: **Physical activity and cardiovascular risk factors in children: meta-analysis of randomized clinical trials.** *Prev Med (Baltim)* 2014, **69**:54–62.

21. Leon AS, Sanchez OA: **Response of blood lipids to exercise training alone or combined with dietary intervention.** *Med Sci Sports Exerc* 2001, **33**(6 Suppl):S502-15–9.

22. Fagard RH: **Exercise characteristics and the blood pressure response to dynamic physical training.** *Med Sci Sports Exerc* 2001, **33**(6 Suppl):S484-92–4.

23. Law MR, Wald NJ, Rudnicka AR: **Quantifying effect of statins on low density lipoprotein cholesterol, ischaemic heart disease, and stroke: systematic review and meta-analysis.** *BMJ* 2003, **326**:1423.

24. Thompson AM: **Antihypertensive Treatment and Secondary Prevention of Cardiovascular Disease Events Among Persons Without Hypertension**. *JAMA* 2011, **305**:913.

25. Tuomilehto J, Lindström J, Eriksson JG, Valle TT, Hämäläinen H, Ilanne-Parikka P, Keinänen-Kiukaanniemi S, Laakso M, Louheranta A, Rastas M, Salminen V, Uusitupa M: **Prevention of type 2 diabetes mellitus by changes in lifestyle among subjects with impaired glucose tolerance.** *N Engl J Med* 2001, **344**:1343–50.

26. Pan XR, Li GW, Hu YH, Wang JX, Yang WY, An ZX, Hu ZX, Lin J, Xiao JZ, Cao HB, Liu PA, Jiang XG, Jiang YY, Wang JP, Zheng H, Zhang H, Bennett PH, Howard B V: **Effects of diet and exercise in preventing NIDDM in people with impaired glucose tolerance. The Da Qing IGT and Diabetes Study.** *Diabetes Care* 1997, **20**:537–44.

27. Pirie K, Peto R, Reeves GK, Green J, Beral V: **The 21st century hazards of smoking and benefits of stopping: a prospective study of one million women in the UK.** *Lancet* 2013, **381**:133–41.

28. Pinsky PF, Zhu CS, Kramer BS: **Lung cancer risk by years since quitting in 30+ pack year smokers.** *J Med Screen* 2015.

29. Wakai K, Marugame T, Kuriyama S, Sobue T, Tamakoshi A, Satoh H, Tajima K, Suzuki T, Tsugane S: **Decrease in risk of lung cancer death in Japanese men after smoking cessation by age at quitting: pooled analysis of three large-scale cohort studies.** *Cancer Sci* 2007, **98**:584–9.

30. Harriss DJ, Atkinson G, Batterham A, George K, Cable NT, Reilly T, Haboubi N, Renehan AG: **Lifestyle factors and colorectal cancer risk (2): a systematic review and meta-analysis of associations with leisure-time physical activity.** *Colorectal Dis* 2009, **11**:689–701.

31. Monninkhof EM, Elias SG, Vlems FA, van der Tweel I, Schuit AJ, Voskuil DW, van Leeuwen FE: **Physical activity and breast cancer: a systematic review.** *Epidemiology* 2007, **18**:137–57.

32. Whalley LJ, Dick FD, McNeill G: **A life-course approach to the aetiology of late-onset dementias.** *Lancet Neurol* 2006, **5**:87–96.

33. **A two-decade comparison of prevalence of dementia in individuals aged 65 years and older from three geographical areas of England: results of the Cognitive Function and Ageing Study I and II - PIIS0140673613615706.pdf** [http://download.thelancet.com/pdfs/journals/lancet/PIIS0140673613615706.pdf?id=410a13c7e856fa01:-537181ea:142b8fe4bec:1b371386088006979]

34. Schrijvers EMC, Verhaaren BFJ, Koudstaal PJ, Hofman A, Ikram MA, Breteler MMB: **Is dementia incidence declining?: Trends in dementia incidence since 1990 in the Rotterdam Study.** *Neurology* 2012, **78**:1456–63.

35. Schmid D, Leitzmann MF: **Association between physical activity and mortality among breast cancer and colorectal cancer survivors: a systematic review and meta-analysis.** *Ann Oncol* 2014, **25**:1293–1311.

36. **Metrics: Population Attributable Fraction (PAF)** [http://www.who.int/healthinfo/global_burden_disease/metrics_paf/en/]

37. Hamer M, Chida Y: **Active commuting and cardiovascular risk: a meta-analytic review**. *Prev Med* 2008, **46**:9–13.

38. Jeon CY, Lokken RP, Hu FB, van Dam RM: **Physical activity of moderate intensity and risk of type 2 diabetes: a systematic review.** *Diabetes Care* 2007, **30**:744–52.

39. Hamer M, Chida Y: **Physical activity and risk of neurodegenerative disease: a systematic review of prospective evidence**. *Psychol Med* 2009, **39**:3–11.

40. O’Rorke MA, Cantwell MM, Cardwell CR, Mulholland HG, Murray LJ: **Can physical activity modulate pancreatic cancer risk? a systematic review and meta-analysis.** *Int J Cancer* 2010, **126**:2957–68.

41. Liu Y, Hu F, Li D, Wang F, Zhu L, Chen W, Ge J, An R, Zhao Y: **Does physical activity reduce the risk of prostate cancer? A systematic review and meta-analysis.** *Eur Urol* 2011, **60**:1029–44.

42. Heran BS, Chen JM, Ebrahim S, Moxham T, Oldridge N, Rees K, Thompson DR, Taylor RS: **Exercise-based cardiac rehabilitation for coronary heart disease.** *Cochrane database Syst Rev* 2011:CD001800.

43. Office for National Statistics: *Deaths Registered in England and Wales, 2011*. Newport, Wales; 2013.

44. Office for National Statistics: *Cancer Registrations in England, 2011*. Newport, Wales; 2013.

45. Office for National Statistics: *Mortality Statistics: Deaths Registered in England and Wales, 2012*. London; 2014.

46. National Centre for Social Research: *Health Survey for England, 2012 [Computer File]*. Colchester; 2013.

47. Health & Social Care Information Centre: *National Diabetes Audit 2011-12 Report 2: Complications & Mortality*. Leeds; 2013.

48. Rait G, Walters K, Bottomley C, Petersen I, Iliffe S, Nazareth I: **Survival of people with clinical diagnosis of dementia in primary care: cohort study.** *BMJ* 2010, **341**:c3584.
