# Supplementary material for: The modelled impact of increases in physical activity: the effect of both increased survival and reduced incidence of disease
Source: Eur J Epidemiol. 2017 Mar 3;32(3):235–50. doi: 10.1007/s10654-017-0235-1 (PMC5380706; doi:10.1007/s10654-017-0235-1)
Supplement: Supplementary file 3 — Supplementary material 3 (DOCX 267 kb) [file 10654_2017_235_MOESM3_ESM.docx]

Figure A2: Tornado plot showing the effect of parametric uncertainty on estimates of change in life expectancy (baseline vs 'all adults meeting PA guidelines')

Stroke/IHD = relative risk of stroke incidence and ischaemic heart disease incidence for physical activity; Dementia = relative risk of dementia incidence for physical activity; Colon Ca (m) = relative risk of colon cancer incidence amongst men for physical activity; Colon Ca (w) = relative risk of colon cancer incidence amongst women for physical activity; Breast Ca = relative risk of breast cancer incidence amongst women for physical activity; Diabetes = relative risk of type 2 diabetes incidence for physical activity; IHD CFR = relative risk of mortality amongst people with diagnosed IHD for physical activity; Breast Ca CFR = relative risk of mortality amongst women with diagnosed breast cancer for physical activity; Colon Ca CFR = relative risk of mortality for people with diagnosed colon cancer for physical activity; Dose response inc = log linear power transformation for association between physical activity and disease incidence; Dose response CFR = log linear power transformation for association between physical activity and mortality from disease (either IHD, colon cancer or breast cancer); the range of values used for each parameter are shown in the methods supplement (Table A3); baseline = no change in physical activity; ‘All adults meeting PA guidelines’ scenario assumes that all adults who are not presently doing 5.75 marginal MET-hours of physical activity (equivalent to 150 minutes of walking at 3mph on flat ground (3.3 MET) per week) increase their physical activity to 5.75 marginal MET-hours, the physical activity level of adults who are doing more than 5.75 marginal MET-hours per week is unchanged. Tornado plots are a special type of bar chart, where the bars are arranged horizontally and in order of bar size, typically with the largest bar at the top and the smallest bar at the bottom so that the diagram forms a visual ‘tornado’. Tornado plots are a common means to undertake ‘deterministic sensitivity analyses’, where the relative importance of variation in different parameters is compared. Used in this way each bar represents the range of outcome values expected consistent with the reported or described uncertainty for the given input. The inputs whose uncertainty contributes most to uncertainty in the outcome will have the largest bars and thus be at the top of the diagram. The tornado plot is centred around the mid-point (median) estimate for all parameters.

Figure A3: Tornado plots showing the effect of parametric uncertainty on estimates of change in person-years lived with disease (baseline vs ‘all adults meeting PA guidelines’)


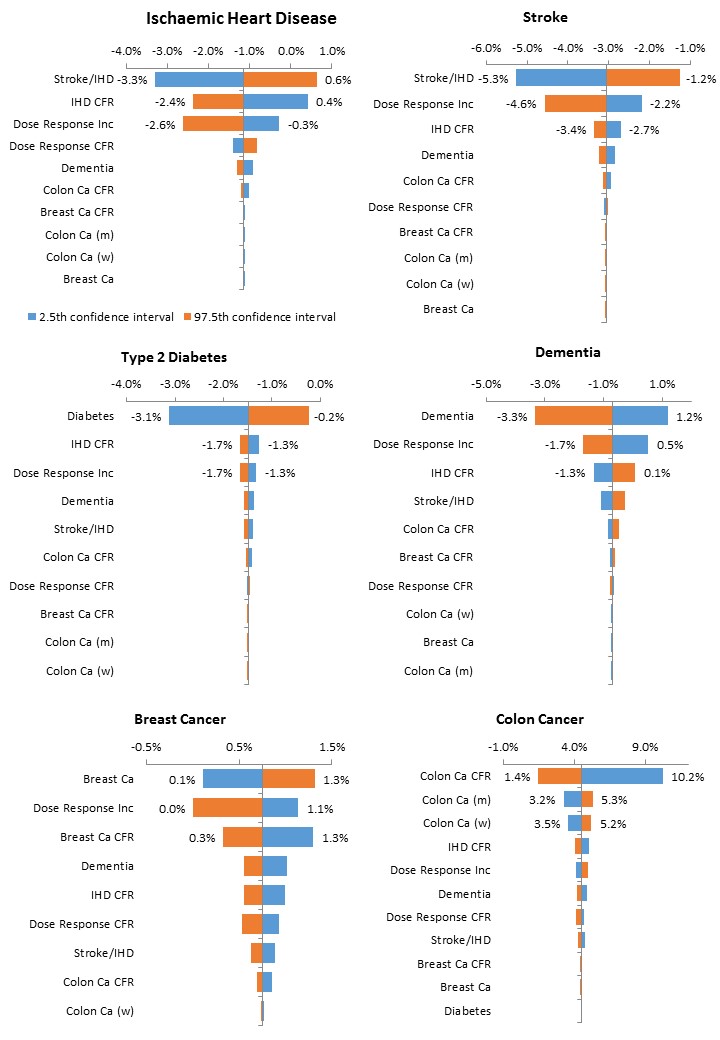
**Figure A4: Tornado plots showing the effect of parametric uncertainty on estimates of change in incident cases (baseline vs ‘all adults meeting PA guidelines’)**


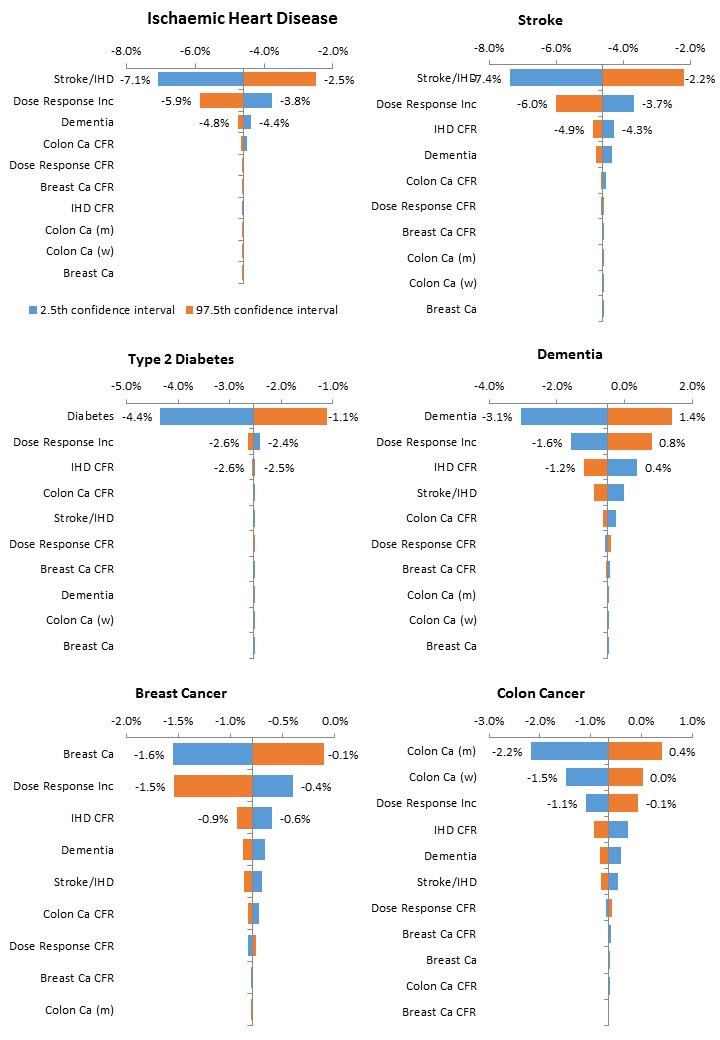


**Footnote for Figure A3 and Figure A4**

Stroke/IHD = relative risk of stroke incidence and ischaemic heart disease incidence for physical activity; Dementia = relative risk of dementia incidence for physical activity; Colon Ca (m) = relative risk of colon cancer incidence amongst men for physical activity; Colon Ca (w) = relative risk of colon cancer incidence amongst women for physical activity; Breast Ca = relative risk of breast cancer incidence amongst women for physical activity; Diabetes = relative risk of type 2 diabetes incidence for physical activity; IHD CFR = relative risk of mortality amongst people with diagnosed IHD for physical activity; Breast Ca CFR = relative risk of mortality amongst women with diagnosed breast cancer for physical activity; Colon Ca CFR = relative risk of mortality for people with diagnosed colon cancer for physical activity; Dose response inc = log linear power transformation for association between physical activity and disease incidence; dose response CFR = log linear power transformation for association between physical activity and mortality from disease (either IHD, colon cancer or breast cancer); the range of values used for each parameter are shown in the methods supplement (Table A3); baseline = no change in physical activity; ‘All adults meeting PA guidelines’ scenario assumes that all adults who are not presently doing 5.75 marginal MET-hours of physical activity (equivalent to 150 minutes of walking at 3mph on flat ground (3.3 MET) per week) increase their physical activity to 5.75 marginal MET-hours, the physical activity level of adults who are doing more than 5.75 marginal MET-hours per week is unchanged. Tornado plots are a special type of bar chart, where the bars are arranged horizontally and in order of bar size, typically with the largest bar at the top and the smallest bar at the bottom so that the diagram forms a visual ‘tornado’. Tornado plots are a common means to undertake ‘deterministic sensitivity analyses’, where the relative importance of variation in different parameters is compared. Used in this way each bar represents the range of outcome values expected consistent with the reported or described uncertainty for the given input. The inputs whose uncertainty contributes most to uncertainty in the outcome will have the largest bars and thus be at the top of the diagram. The tornado plot is centred around the mid-point (median) estimate for all parameters.

**Figure A4: Tornado plot to show the effect of parametric uncertainty on change in incident cases for the six diseases**

|  |  |
| --- | --- |
|  |  |
|  |  |

Stroke/IHD = relative risk of stroke incidence and ischaemic heart disease incidence for physical activity; Dementia = relative risk of dementia incidence for physical activity; Colon Ca (m) = relative risk of colon cancer incidence amongst men for physical activity; Colon Ca (w) = relative risk of colon cancer incidence amongst women for physical activity; Breast Ca = relative risk of breast cancer incidence amongst women for physical activity; Diabetes = relative risk of type 2 diabetes incidence for physical activity;IHD CFR = relative risk of mortality amongst people with diagnosed IHD for physical activity; Breast Ca CFR = relative risk of mortality amongst women with diagnosed breast cancer for physical activity; Colon Ca CFR = relative risk of mortality for people with diagnosed colon cancer for physical activity; DR shape (inc) = log linear power transformation for association between physical activity and disease incidence; DR shape (CFR) = log linear power transformation for association between physical activity and mortality from disease (either IHD, colon cancer or breast cancer); Shift (225) represents a scenario of everyone doing an additional 8.675 marginal MET-hours of physical activity per week, equivalent to an additional 225 minutes of additional moderate physical activity at 3.3 MET, e.g. walking at 3mph on level ground.
